# Protective role of endogenous cardiac natriuretic peptide/GC-A system on the maternal heart during the postpartum period

**DOI:** 10.1186/2050-6511-14-S1-P53

**Published:** 2013-08-29

**Authors:** Kentaro Otani, Takeshi Tokudome, Ichiro Kishimoto, Yuanjie Mao, Kenichi Yamahara, Kazuwa Nakao, Kenji Kangawa

**Affiliations:** 1 Department of Regenerative Medicine and Tissue Engineering, National Cerebral and Cardiovascular Center Research Institute, Osaka, Japan; 2 Department of Biochemistry, National Cerebral and Cardiovascular Center Research Institute, Osaka, Japan; 3 Department of Medicine and Clinical Science, Kyoto University Graduate School of Medicine, Kyoto, Japan

## 

**Figure 1:**
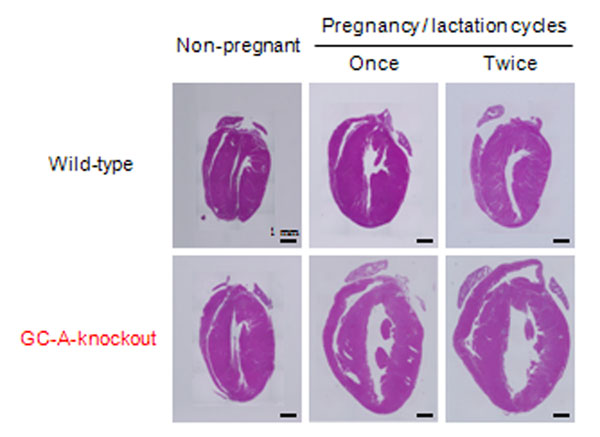
Significant cardiac hypertrophy was observed in GC-A-knockout mice during the puerperal period.

Volume overload during pregnancy results in transient and reversible cardiac hypertrophy in pregnant females. Endogenous atrial natriuretic peptide (ANP) and brain natriuretic peptide (BNP) acts through the common receptor, guanylyl cyclase-A (GC-A) to lower blood pressure, induce diuresis/natriuresis, and dilate blood vessels. Although the roles of endogenous natriuretic peptides/GC-A system on the cardiovascular system have been well investigated, those on the puerperal period have not been elucidated. The aim of this study was to investigate the physiological role of endogenous natriuretic peptides/GC-A system in the puerperal period by comparing pregnant wild-type (WT) and GC-A knockout (GC-A-KO) mice. Although blood pressure in GC-A-KO mice was not changed during pregnancy or after delivery, there was a dramatic postpartum increase in cardiac weight. Interestingly, the cardiac hypertrophy and interstitial fibrosis in GC-A-KO were dramatically accelerated by repeated pregnancy/lactation cycles (Figure 1). Moreover, a significant decline in cardiac function, as well as up-regulation of fetal cardiac genes, was observed in the multiparous GC-A-KO heart. Importantly, the hypertrophic change in GC-A-KO heart was observed not during gestation, but during lactation. Furthermore, the lactation-induced cardiac hypertrophy in GC-A-KO was significantly suppressed by avoiding the breast feeding. Our results suggest that the defect in endogenous cardiac natriuretic peptide/GC-A system promotes cardiomyopathy during puerperal period, especially during lactation.

